# Prognostic Value of the Gustave Roussy Immune Score in Patients with Locally Advanced Gastric Cancer Receiving Neoadjuvant FLOT Chemotherapy: A Retrospective Cohort Study

**DOI:** 10.3390/diagnostics16121759

**Published:** 2026-06-07

**Authors:** Aykut Turhan, Mehmet Emin Büyükbayram, Zekeriya Hannarici, Alperen Akansel Çağlar, Yasin Emrah Soylu, Mehmet Bilici, Salim Başol Tekin

**Affiliations:** 1 Department of Medical Oncology, Ordu University Training and Research Hospital, Ordu 52200, Turkey; 2 Department of Medical Oncology, Yalova Training and Research Hospital, Yalova 77200, Turkey; 3 Department of Medical Oncology, Bursa City Hospital, Bursa 16250, Turkey; 4 Department of Medical Oncology, Istanbul Çam and Sakura City Hospital, Istanbul 34480, Turkey; 5 Department of Medical Oncology, Atatürk University Faculty of Medicine, Erzurum 25040, Turkey; 6 Department of Medical Oncology, Acıbadem Bursa Hospital, Bursa 16210, Turkey

**Keywords:** gastric cancer, GRIm score, neoadjuvant chemotherapy, FLOT, overall survival, progression-free survival

## Abstract

**Background:** Gastric cancer remains a leading cause of cancer mortality, and additional prognostic tools reflecting host inflammation and nutritional status are needed, particularly in patients receiving neoadjuvant regimens such as fluorouracil, leucovorin, oxaliplatin, and docetaxel (FLOT). This study aimed to evaluate the prognostic significance of the Gustave Roussy Immune (GRIm) score in patients with locally advanced gastric cancer treated with neoadjuvant FLOT. **Methods:** This retrospective single-center study included 128 patients with locally advanced gastric or gastroesophageal junction (GEJ) adenocarcinoma treated with neoadjuvant FLOT between 2018 and 2022. The GRIm score, based on albumin, lactate dehydrogenase (LDH), and neutrophil-to-lymphocyte ratio (NLR), was used to stratify patients into low- and high-risk groups. Overall survival (OS) and progression-free survival (PFS) were analyzed using Kaplan–Meier and Cox proportional hazards regression models. **Results:** Among 128 patients (mean age, 61.5 years; 63.3% male), 69.5% underwent surgical resection, and 24.7% achieved a pathological complete response. During a median follow-up of 36.9 months, 57.0% of patients experienced disease progression and 48.4% died. The median OS and PFS were 49.9 and 25.5 months, respectively. The GRIm score demonstrated moderate discriminative ability for mortality (area under the curve [AUC]: 0.704) and was significantly associated with survival. The median OS was shorter in the high-risk group than in the low-risk group (35.2 vs. 69.6 months, *p* < 0.001), and the median PFS was also shorter in the high-risk group (17.2 months vs. not reached, *p* < 0.001). In the multivariate analysis, high GRIm risk remained independently associated with worse OS (hazard ratio [HR]: 1.832, *p* = 0.030) and PFS (HR: 2.491, *p* < 0.001), along with age, clinical stage, and surgical resection status. **Conclusions:** In this single-center retrospective cohort study, the GRIm score was associated with survival outcomes in patients with locally advanced gastric or GEJ adenocarcinoma receiving neoadjuvant FLOT. These findings suggest that the GRIm score may provide additional risk stratification when interpreted in conjunction with established clinical factors. However, external validation in larger prospective cohorts is required before it can be applied in routine clinical practice.

## 1. Introduction

Gastric cancer continues to represent a major challenge to global health and is among the most frequently diagnosed cancers and leading cause of cancer-related deaths worldwide [1]. In 2022, approximately 968,000 new cases and 660,000 deaths were reported globally, with the highest incidence rates in East Asia and some regions of Europe [2]. Although multidisciplinary treatment approaches have improved, the prognosis of patients with locally advanced gastric cancer remains poor, and many patients are diagnosed at an advanced stage or develop recurrence after curative-intent surgery [3,4].

The identification of reliable prognostic biomarkers is crucial for improving treatment stratification and patient-tailored care to improve patient outcomes. The tumor–node–metastasis (TNM) staging system is the clinical gold standard; however, it does not consider the biological heterogeneity of tumors and physiological responses of the body [5]. Therefore, there is an urgent need for readily available biomarkers that can provide further prognostic information to the standard clinicopathological criteria [6].

Recent studies have highlighted the importance of systemic inflammation and nutritional status as major determinants of cancer progression and patient prognosis [4,7]. The systemic inflammatory response encourages the growth of tumor cells, epithelial–mesenchymal transition, and immune evasion [5]. In particular, the neutrophil-to-lymphocyte ratio (NLR) is well recognized as a powerful indicator of the balance between tumor-promoting inflammation and tumor-fighting immunity [4]. Serum albumin levels reflect nutritional status and degree of systemic inflammation, and low albumin levels are independently associated with worse survival outcomes [7,8]. Moreover, lactate dehydrogenase (LDH) is a marker of tumor burden and aggressive tumor features, reflecting augmented glycolytic activity and tumor hypoxia, and has been consistently associated with adverse clinical outcomes in various malignancies [9,10].

The Gustave Roussy Immune (GRIm) score is a new composite score that combines LDH, serum albumin, and NLR into a single prognostic index [11,12,13]. Initially developed to better select patients for early-stage clinical trials, the GRIm score provides a comprehensive assessment combining measures of tumor load, host nutritional status, and systemic inflammation [14,15]. Recent meta-analyses and retrospective studies have confirmed the prognostic value of the GRIm score in various solid tumors, including colorectal cancer, esophageal squamous cell carcinoma, and non-small cell lung cancer [11,14,16,17].

The fluorouracil, leucovorin, oxaliplatin, and docetaxel (FLOT) regimen, which includes fluorouracil, leucovorin, oxaliplatin, and docetaxel, has recently been established as the standard treatment for resectable locally advanced gastric and gastroesophageal junction (GEJ) adenocarcinomas [18,19]. The pivotal FLOT4 trial highlighted notable enhancements in overall survival and pathological response rates compared with older regimens based on epirubicin [20,21,22]. Currently, there is a scarcity of evidence concerning the prognostic relevance of the GRIm score in patients undergoing neoadjuvant FLOT chemotherapy. Thus, the present study aimed to evaluate the prognostic significance of the GRIm score in patients with locally advanced gastric cancer undergoing neoadjuvant FLOT chemotherapy.

## 2. Materials and Methods

### 2.1. Study Design and Patients

This retrospective cohort study, conducted at a single center, aimed to assess the prognostic significance of the GRIm score in individuals with locally advanced gastric/GEJ adenocarcinoma who underwent neoadjuvant FLOT chemotherapy at a tertiary referral institution. This study included patients treated at the Department of Medical Oncology, Atatürk University Faculty of Medicine, Erzurum, Turkey, between January 2018 and December 2022.

**Figure 1:**
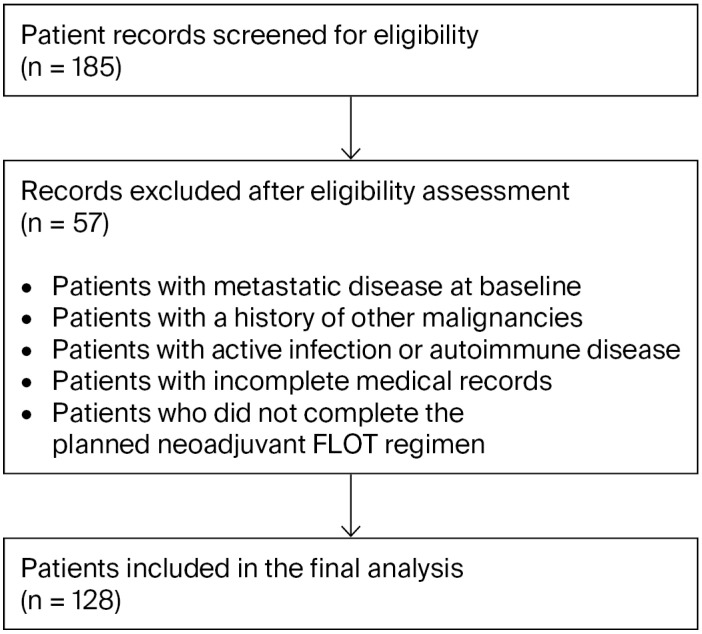
Flow diagram of patient selection and study inclusion.

All patients received a standard neoadjuvant chemotherapy protocol comprising four cycles of FLOT chemotherapy. Prior to treatment initiation, all patients underwent contrast-enhanced thoracoabdominal computed tomography (CT) or positron emission tomography–computed tomography (PET-CT), as well as upper gastrointestinal endoscopy, to confirm locally advanced disease and exclude distant metastases. Patients with metastatic disease on initial imaging were excluded. Eligible patients had an Eastern Cooperative Oncology Group (ECOG) performance status of 0–1, histologically confirmed locally advanced gastric or GEJ adenocarcinoma, including signet ring cell carcinoma, and complete clinical and laboratory data available for analysis. The exclusion criteria were as follows: metastatic disease at diagnosis, history of another malignancy, active infection or autoimmune disease that could affect systemic inflammation, incomplete medical records, and failure to complete neoadjuvant FLOT chemotherapy. A total of 128 patients met the inclusion criteria and were included in the final analysis. Figure 1 illustrates the patient selection process.

### 2.2. Treatment Protocol

All patients were treated with the standard FLOT regimen, which included docetaxel (50 mg/m^2^), oxaliplatin (85 mg/m^2^), leucovorin (200 mg/m^2^), and a continuous 24 h infusion of 5-fluorouracil (2600 mg/m^2^) administered every two weeks. Patients with clinically staged locally advanced disease, characterized as cT2 or higher and/or node-positive (cN+) tumors without signs of distant metastases, received neoadjuvant chemotherapy. Prior to surgical evaluation, all patients completed four cycles of neoadjuvant FLOT chemotherapy at our institution.

The radiological response to neoadjuvant chemotherapy was evaluated using the Response Evaluation Criteria in Solid Tumors (RECIST) criteria, version 1.1. After completing neoadjuvant treatment, a multidisciplinary team reassessed the patients for surgery with curative intent, considering radiological results and clinical status. Surgical resection was planned approximately 4–6 weeks after the completion of neoadjuvant chemotherapy in patients who were deemed operable. Curative-intent gastrectomy (total or subtotal) with lymph node dissection was performed according to tumor location, institutional practice, and contemporary gastric cancer surgical guidelines whenever feasible [23,24]. The number of retrieved lymph nodes, resection status (R0/R1), and pathological response, including pathological complete response (pCR), were assessed by histopathological examination. R0 resection was defined as a microscopically margin-negative resection.

Adjuvant FLOT chemotherapy was administered at the discretion of the treating physician according to postoperative recovery and performance status and was not included as a criterion for patient selection in this study.

### 2.3. Pathological Evaluation

Experienced pathologists evaluated all surgical specimens according to standardized pathological protocols, and all assessments were performed by the same dedicated pathology team to ensure the consistency of the results. Pretreatment clinical staging was performed according to the American Joint Committee on Cancer (AJCC) clinical tumor–node–metastasis (cTNM) classification system, 8th edition. Post-treatment pathological staging was determined according to the AJCC pathological tumor–node–metastasis classification after neoadjuvant chemotherapy (ypTNM). pCR was defined as the absence of residual invasive tumors in both the primary tumor site and regional lymph nodes. Patients with residual disease were classified according to their post-treatment pathological tumor (ypT) and nodal (ypN) stages.

### 2.4. Data Collection and Definitions

Data from clinical and laboratory sources were retrospectively gathered from the electronic medical records. The variables included demographic and clinical details such as age, sex, ECOG performance status, clinical stage, histological subtype, and resection status (R0/R1). Baseline laboratory metrics, such as serum albumin, LDH, and absolute neutrophil and lymphocyte counts, were documented within two weeks prior to the start of neoadjuvant chemotherapy. NLR was determined by dividing the absolute neutrophil count by the absolute lymphocyte count.

The GRIm score was calculated based on three parameters: serum albumin < 3.5 g/dL, LDH above the upper limit of normal (≥240 U/L), and NLR ≥ 6. Each abnormal parameter was assigned one point, whereas normal values were assigned zero points, in accordance with the original GRIm score definition and previous studies [11,25]. Patients were subsequently categorized into low- (score 0–1) and high-risk (score 2–3) groups.

### 2.5. Outcome Measures

The primary endpoints of this study were overall survival (OS) and progression-free survival (PFS). OS was defined as the time from the date of diagnosis to death from any cause or the last follow-up date. PFS was defined as the time from the initiation of neoadjuvant chemotherapy to documented disease progression, recurrence, or death from any cause, whichever occurred first. Patients without events were censored at their last follow-up.

### 2.6. Statistical Analysis

Data analysis was conducted using IBM SPSS Statistics version 31.0 (IBM Corp., Armonk, NY, USA). Continuous variables are presented as mean ± standard deviation (SD) or median (interquartile range [IQR]), depending on the data distribution, whereas categorical variables are presented as frequencies. The baseline clinicopathological characteristics of the GRIm low- and high-risk groups were compared using the independent samples t-test, Mann–Whitney U test, chi-square test, or Fisher’s exact test, as appropriate. Receiver operating characteristic (ROC) curve analysis was performed to evaluate the discriminative ability of the NLR and the predefined GRIm risk classification for mortality. The area under the curve (AUC) was calculated for each parameter. The exploratory cutoff value for NLR was determined using the Youden index, whereas the GRIm score was analyzed according to the predefined low- and high-risk categories described in previous studies.

OS and PFS were estimated using the Kaplan–Meier method, and differences between the groups were compared using the log-rank test. Univariate and multivariate Cox proportional hazards regression analyses were performed to identify prognostic factors associated with survival outcomes. Variables with a *p*-value < 0.10 in the univariate analysis, together with clinically relevant variables, were included in the multivariate models. Hazard ratios (HRs) with 95% confidence intervals (CIs) were reported.

The discriminative performance of the multivariate Cox regression models was assessed using Harrell’s concordance index (C-index). Model calibration for 3-year OS and PFS was evaluated by comparing model-predicted survival probabilities with Kaplan–Meier-estimated observed survival probabilities across the quartiles of predicted risk. Decision curve analysis (DCA) was performed to assess the potential clinical net benefits of the prognostic models across a range of threshold probabilities. The full model, which included GRIm risk classification together with clinically relevant variables, was compared with the clinical model without GRIm, GRIm alone, and treat-all and treat-none strategies. Exploratory nomograms were constructed based on the final multivariable Cox regression models to visualize individualized 3-year OS and PFS probabilities, and are presented in the Supplementary Material.

Given the retrospective single-center design and lack of external validation, calibration curves, decision curve analyses, and nomograms were considered exploratory model performance analyses. A two-sided *p*-value < 0.05 was considered statistically significant.

### 2.7. Ethical Approval

The study was approved by the Clinical Research Ethics Committee of Atatürk University Faculty of Medicine, Erzurum, Turkey (Approval No. B.30.2.ATA.0.01.00/36, Date: 29 December 2022). Owing to the retrospective design of the study, the requirement for informed consent was waived. This study was conducted in accordance with the principles of the Declaration of Helsinki.

### 2.8. Language Editing Statement

AI-assisted language editing tools (Paperpal, version 4.15.5; ChatGPT, version 5.3) were used solely for language refinement, including grammar correction, paraphrasing, and translation support during the final proofreading stage. The authors take full responsibility for the content and integrity of the manuscript.

## 3. Results

### 3.1. Baseline Clinical and Pathological Characteristics

The study included 128 patients with locally advanced gastric or GEJ adenocarcinoma who received neoadjuvant FLOT chemotherapy. The mean age was 61.46 ± 9.01 years (range: 36–81 years), and 63.3% of patients were male. A history of smoking was observed in 41.4% of patients. Patients had a good performance status, with 21.9% having an ECOG score of 0 and 78.1% having an ECOG score of 1. Comorbidities were observed in 38.3% of patients, with hypertension being the most common, followed by coronary artery disease and diabetes mellitus.

Adenocarcinoma was the predominant histological subtype (56.3%), while signet-ring cell carcinoma accounted for 43.8% of cases. The most frequent tumor location was the gastric body (68.0%), followed by the antrum/pylorus (18.0%) and gastroesophageal junction (14.1%). At diagnosis, most patients present with locally advanced disease. The most common clinical T stage was T3 (64.8%), followed by T2 (22.7%) and T4a (10.9%) stages. Lymph node involvement was frequent, with N2 (25.0%) and N3 (22.7%) involvement being the most common. At diagnosis, 8.6% of the patients were at stage I, 35.9% at stage II, 53.9% at stage III, and 1.6% at stage IVA disease. Following neoadjuvant chemotherapy, 69.5% of patients (*n* = 89) underwent surgical resection. Among the 39 patients who did not undergo surgical resection, the reasons included deterioration in performance status, treatment-related toxicity, comorbid conditions precluding surgery, patient refusal, and disease progression during the neoadjuvant treatment. In the resected patients, pCR was achieved in 24.7% of the cases. The most common post-treatment tumor stage was ypT3 (40.4%), and 58.4% of the patients achieved ypN0 status, indicating nodal downstaging. According to postoperative staging, 14.6% of the patients were stage I, 28.1% were stage II, and 32.6% were stage III.

Baseline laboratory parameters showed a mean neutrophil count of 5.04 ± 1.91 × 10^9^/L and a mean lymphocyte count of 2.15 ± 0.75 × 10^9^/L, resulting in a mean NLR of 2.75 ± 1.82. The mean lactate LDH level was 233.25 ± 63.69 U/L, and the mean serum albumin level was 3.81 ± 0.39 g/dL. Based on the GRIm score, 50.8% of patients had a score of 0, 20.3% had a score of 1, 24.2% had a score of 2, and 4.7% had a score of 3. When categorized into risk groups, 71.1% of patients were classified as low-risk and 28.9% as high-risk.

The median follow-up duration was 36.9 months (IQR: 30.4–49.0 months). During the follow-up period, 57.0% of the patients experienced disease progression, and 48.4% died. A detailed summary of the baseline clinicopathological characteristics is presented in Table 1, Table 2 and Table 3.

### 3.2. Baseline Comparison According to GRIm Risk Groups

Baseline demographic and clinicopathological characteristics were compared between the GRIm low-risk and high-risk GRIm groups. Most baseline variables, including sex, ECOG performance status, comorbidity status, histological subtype, tumor location, clinical T stage, and surgical resection status, were generally balanced between groups. Although patients in the high-risk GRIm group tended to present with more advanced nodal involvement and clinical stage, these differences were not statistically significant. A detailed comparison between the groups is presented in Table 4.

### 3.3. ROC Analysis

To assess the predictive performance of the NLR and GRIm scores for mortality, ROC curve analysis was performed. The GRIm score demonstrated moderate discriminative ability (AUC: 0.704), whereas the NLR showed limited discriminative performance (AUC: 0.530).

Using the Youden index, the exploratory ROC-derived cutoff value for the GRIm score was identified as 1.5, which was consistent with the predefined GRIm risk classification (low-risk: score 0–1; high-risk: score 2–3). Exploratory ROC analysis identified a cohort-specific NLR cutoff value of 3.43. However, despite this cutoff, NLR alone demonstrated a limited discriminative ability. Because the GRIm score is a previously validated composite prognostic score based on predefined thresholds, the original GRIm classification incorporating NLR ≥ 6 was retained for the primary analyses to preserve the methodological consistency and comparability with prior studies [11,25]. Overall, the GRIm score demonstrated superior predictive performance compared with that of NLR alone.

### 3.4. Overall Survival

The median OS for the entire cohort was 49.9 months (95% CI: 43.5–56.3). Kaplan–Meier survival analysis demonstrated that OS did not differ significantly according to sex. The median OS was 49.9 months in male patients and 50.3 months in female patients (log-rank, *p* = 0.667). In contrast, clinical stage was significantly associated with survival outcomes. Patients with stage I–II disease had a substantially longer median OS of 69.6 months compared to 44.7 months in those with stage III–IV disease (log-rank *p* < 0.001). Histopathological subtype was not significantly associated with OS. The median OS was 53.4 months in patients with adenocarcinoma and 48.6 months in those with signet-ring cell carcinoma (log-rank *p* = 0.180).

**Figure 2:**
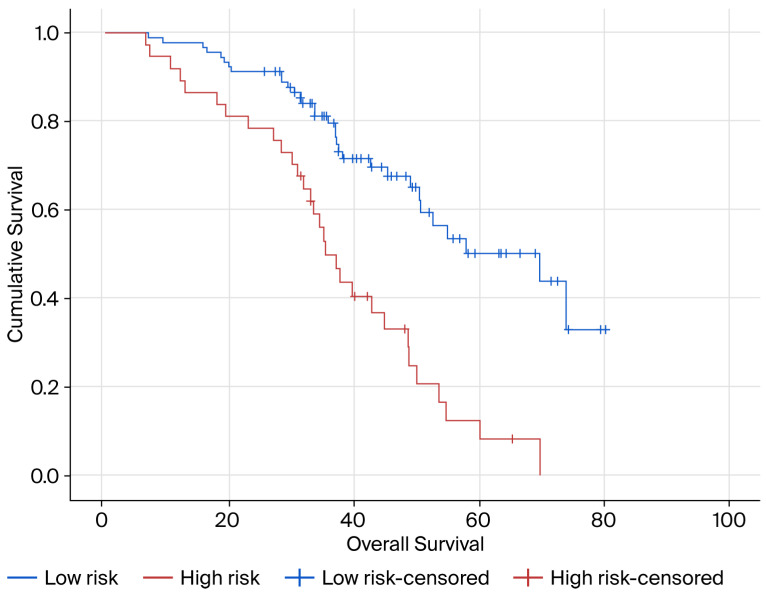
Kaplan–Meier curves for overall survival according to GRIm risk classification. Overall survival was significantly longer in the low-risk group (GRIm score 0–1) than in the high-risk group (GRIm score 2–3). The median overall survival was 69.6 months (95% CI: 46.2–93.0) in the low-risk group and 35.2 months (95% CI: 30.8–39.6) in the high-risk group (log-rank *p* < 0.001).

In exploratory analyses using the cohort-specific NLR cutoff value (3.43), patients with NLR < 3.43 demonstrated a numerically longer median overall survival (50.3 months) than those with NLR ≥ 3.43 (39.5 months); however, this difference did not reach statistical significance (log-rank *p* = 0.183). Notably, the GRIm risk classification was significantly associated with survival. Patients in the low-risk group had a markedly longer median OS of 69.6 months compared to 35.2 months in the high-risk group (log-rank *p* < 0.001), as illustrated in Figure 2. The 3-year OS rate was 79.6% in the GRIm low-risk group and 49.8% in the high-risk group.

Surgical resection was strongly associated with improved survival. Patients who underwent surgery had a median OS of 60.0 months, whereas those who did not undergo surgery had a significantly shorter median OS of 31.2 months (log-rank *p* < 0.001). Kaplan–Meier analysis according to pathological stage after neoadjuvant therapy (ypStage) demonstrated significant differences in overall survival among the resected patients (log-rank *p* < 0.001). Patients who achieved pCR had the most favorable outcomes, with a median OS of 73.8 months (95% CI: 50.9–96.7). The median OS was 69.6 months (95% CI: 42.4–96.9) in ypStage I patients, 53.4 months (95% CI: 50.1–56.7) in ypStage II patients, and 48.6 months (95% CI: 39.0–58.1) in ypStage III patients.

### 3.5. Progression-Free Survival

Kaplan–Meier analysis demonstrated that the median PFS for the entire cohort was 25.5 months (95% CI: 15.7–35.3). No significant difference in PFS was observed according to sex. The median PFS was 25.0 months in male patients and 28.1 months in female patients (log-rank *p* = 0.699). Clinical stage was significantly associated with PFS. Patients with stage I–II disease demonstrated longer progression-free survival compared to those with stage III–IV disease (median PFS: not reached vs. 18.4 months; log-rank *p* = 0.009). In this study, histological subtype was also associated with PFS. Patients with adenocarcinoma had a longer median PFS of 42.9 months, whereas those with signet-ring cell carcinoma had a shorter median PFS of 18.3 months (log-rank *p* = 0.029).

**Figure 3:**
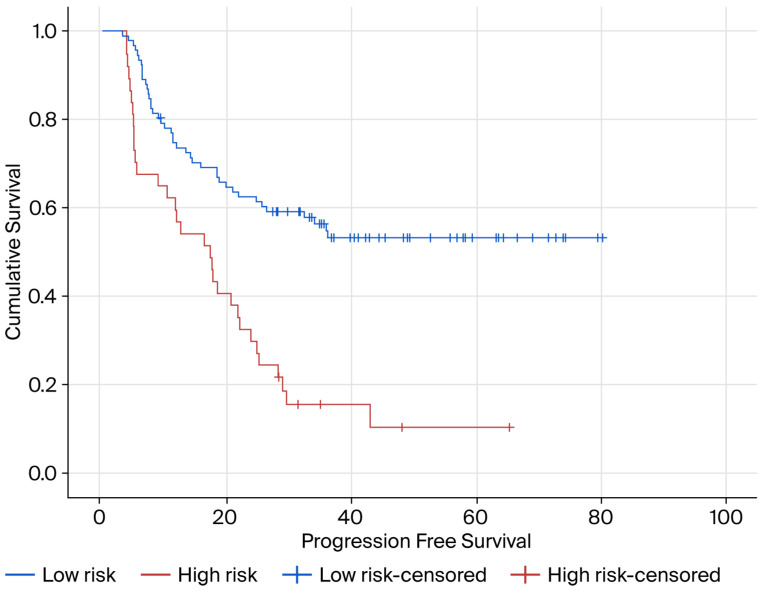
Kaplan–Meier curves for progression-free survival according to GRIm risk classification. Progression-free survival was significantly longer in the low-risk group (GRIm score 0–1) than in the high-risk group (GRIm score 2–3). The median progression-free survival was not reached in the low-risk group, whereas it was 17.2 months (95% CI: 10.3–24.1) in the high-risk group (log-rank *p* < 0.001).

In exploratory analyses using the cohort-specific NLR cutoff value (3.43), patients with NLR < 3.43 demonstrated a numerically longer median progression-free survival (32.4 months) than those with NLR ≥ 3.43 (18.3 months); however, this difference did not reach statistical significance (log-rank *p* = 0.093). Notably, the GRIm score was significantly associated with PFS. Patients in the low-risk group had a markedly longer median PFS than those in the high-risk group (median not reached vs. 17.2 months, log-rank *p* < 0.001), as shown in Figure 3. The 3-year PFS rates were 54.7% and 15.4% in the GRIm low-and high-risk groups, respectively.

Surgical resection was also strongly associated with improved PFS. Patients who underwent surgery had substantially longer progression-free survival than those who did not (median not reached vs. 6.3 months, log-rank *p* < 0.001). Kaplan–Meier analysis according to postoperative ypStage demonstrated significant differences in progression-free survival among the resected patients (log-rank *p* < 0.001). Patients who achieved pCR demonstrated the most favorable PFS outcomes, whereas those with ypStage III disease had the poorest outcomes. Median PFS was not reached in patients with pCR, ypStage I, or ypStage II disease. In contrast, patients with ypStage III disease had a median PFS of 20.8 months (95% CI: 8.6–33.0 months).

### 3.6. Prognostic Factors for Overall Survival

In the univariate Cox regression analysis, age, clinical stage, surgical resection, and GRIm risk classification were significantly associated with overall survival. In contrast, sex, histological subtype, and exploratory cohort-specific NLR category were not significantly associated with OS.

Increasing age was associated with poorer survival (HR: 1.045, 95% CI: 1.013–1.077, *p* = 0.005). Patients with advanced-stage disease (stage III–IV) had significantly worse survival than those with stage I–II disease (HR: 2.619, 95% CI: 1.490–4.605, *p* < 0.001). Surgical resection was strongly associated with improved survival outcomes, with patients undergoing surgery demonstrating a markedly reduced risk of mortality (HR: 0.137, 95% CI: 0.080–0.233, *p* < 0.001). Importantly, a high GRIm risk classification was strongly associated with worse survival (HR: 3.099, 95% CI: 1.869–5.138, *p* < 0.001).

**Figure 4:**
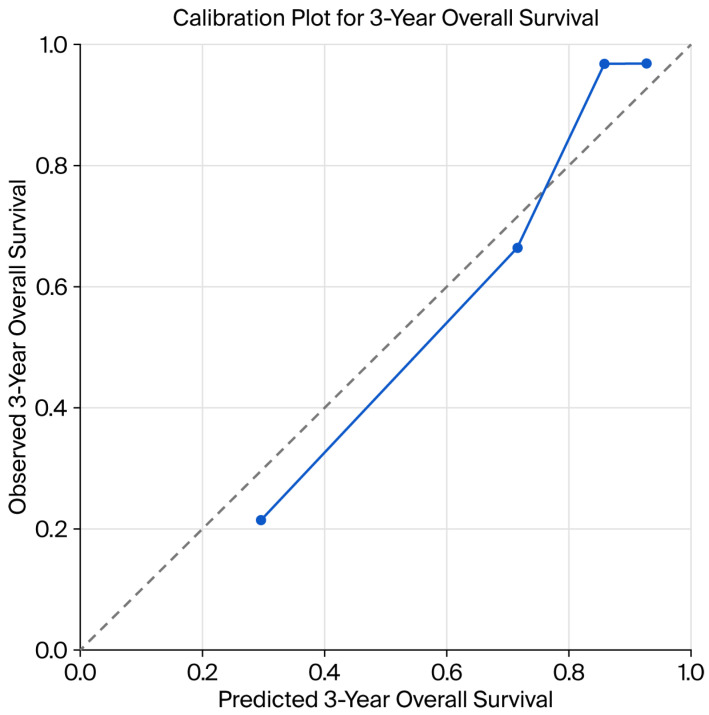
Calibration plot for the 3-year overall survival prediction model. The calibration plot shows the agreement between model-predicted and Kaplan–Meier-estimated observed 3-year overall survival probabilities across quartiles of predicted risk. The dashed diagonal line represents perfect calibration, and the solid line represents the observed survival probability. The model showed acceptable apparent calibration for 3-year overall survival in this cohort.

In the multivariate Cox regression analysis of the overall cohort, age, clinical stage, surgical resection status, and GRIm risk classification were included in the model. Increasing age was independently associated with worse OS (HR: 1.035, 95% CI: 1.007–1.064, *p* = 0.013). Advanced clinical stage was also independently associated with poorer OS (HR: 2.257, 95% CI: 1.258–4.050, *p* = 0.006). Surgical resection was independently associated with improved survival, with patients undergoing surgery showing a reduced risk of mortality (HR: 0.164, 95% CI: 0.095–0.285, *p* < 0.001). High GRIm risk classification remained independently associated with worse OS (HR: 1.832, 95% CI: 1.059–3.169, *p* = 0.030). The overall multivariate model was statistically significant (omnibus test *p* < 0.001) and demonstrated good discriminative performance, with a Harrell’s C-index of 0.831. Calibration analysis for 3-year OS demonstrated acceptable agreement between the model-predicted and Kaplan–Meier-estimated observed survival probabilities across quartiles of predicted risk (Figure 4). The findings are summarized in Table 5.

### 3.7. Prognostic Factors for Progression-Free Survival

In the univariate Cox regression analysis, age, histological subtype, clinical stage, surgical resection, and GRIm risk classification were significantly associated with progression-free survival. In contrast, sex and exploratory cohort-specific NLR category were not significantly associated with PFS.

Increasing age was associated with poorer PFS (HR: 1.029, 95% CI: 1.002–1.058, *p* = 0.038). Patients with advanced-stage disease (stage III–IV) had significantly worse PFS than those with stage I–II disease (HR: 1.896, 95% CI: 1.166–3.081, *p* = 0.010). Histological subtype was also associated with PFS, with signet-ring-cell carcinoma demonstrating poorer outcomes compared to adenocarcinoma (HR: 1.661, 95% CI: 1.048–2.632, *p* = 0.031). Surgical resection was strongly associated with improved PFS, with patients undergoing surgery showing a markedly reduced risk of disease progression (HR: 0.037, 95% CI: 0.020–0.069, *p* < 0.001). Similarly, a high GRIm risk classification was significantly associated with worse PFS (HR: 2.874, 95% CI: 1.798–4.593, *p* < 0.001).

**Figure 5:**
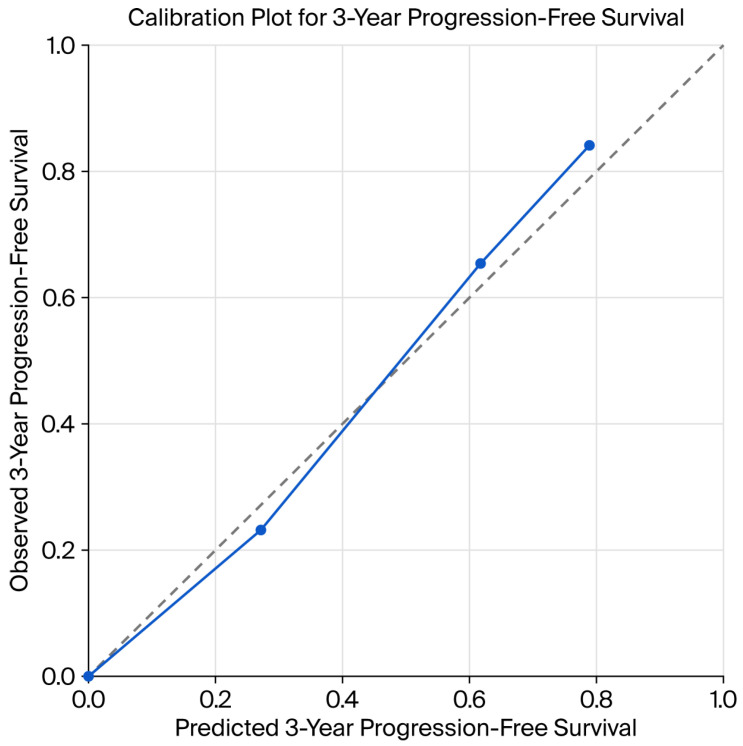
Calibration plot for the 3-year progression-free survival prediction model. The calibration plot shows the agreement between model-predicted and Kaplan–Meier-estimated observed 3-year progression-free survival probabilities across quartiles of predicted risk. The dashed diagonal line represents perfect calibration, and the solid line represents the observed progression-free survival probability. The model showed acceptable apparent calibration for 3-year progression-free survival in this cohort.

In the multivariate Cox regression analysis of the overall cohort, age, histological subtype, clinical stage, surgical resection status, and GRIm risk classification were included in the model. Increasing age was independently associated with worse PFS (HR: 1.050, 95% CI: 1.022–1.078, *p* < 0.001). Advanced clinical stage was also independently associated with poorer PFS (HR: 1.722, 95% CI: 1.028–2.883, *p* = 0.039). Surgical resection was independently associated with improved PFS (HR: 0.039, 95% CI: 0.020–0.076, *p* < 0.001). High-risk GRIm classification remained independently associated with worse PFS (HR: 2.491, 95% CI: 1.483–4.185, *p* < 0.001). Histological subtype showed a trend toward worse PFS for signet-ring-cell carcinoma than for adenocarcinoma (HR: 1.517, 95% CI: 0.926–2.485, *p* = 0.098). The overall multivariate PFS model was statistically significant (omnibus test *p* < 0.001) and demonstrated good discriminative performance, with a Harrell’s C-index of 0.859. Calibration analysis for 3-year PFS demonstrated acceptable agreement between model-predicted and Kaplan–Meier-estimated observed progression-free survival probabilities across quartiles of predicted risk (Figure 5). The findings are summarized in Table 6.

**Figure 6:**
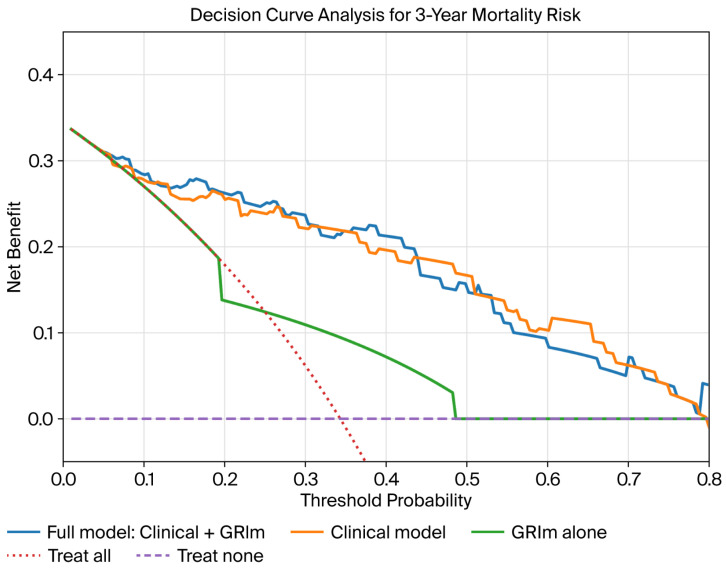
Decision curve analysis of 3-year mortality risk. Decision curve analysis was performed to evaluate the potential clinical utility of the multivariate prediction model for 3-year mortality risk. The full model, incorporating age, clinical stage, surgical resection status, and GRIm risk classification, was compared with the clinical model without GRIm, GRIm alone, and the treat-all and treat-none strategies across a range of threshold probabilities. The full model showed a favorable net benefit across clinically relevant threshold probabilities.

**Figure 7:**
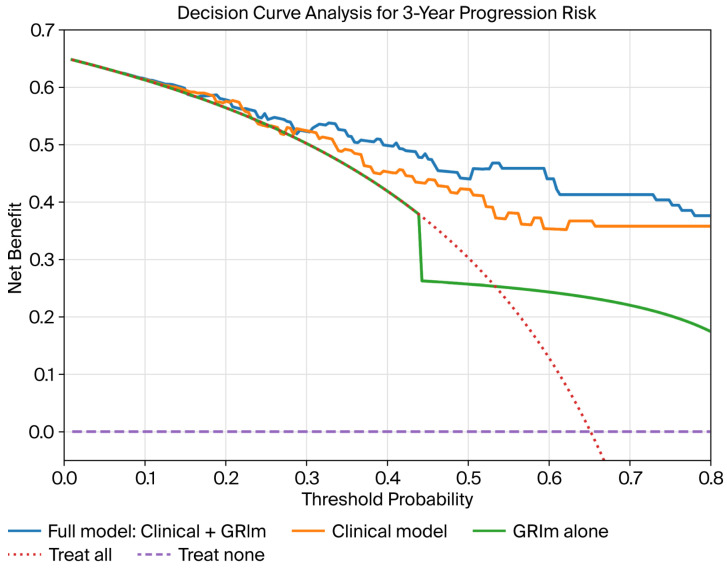
Decision curve analysis for 3-year progression risk. Decision curve analysis was performed to evaluate the potential clinical utility of the prediction model for estimating the 3-year progression risk. The full model, incorporating age, histological subtype, clinical stage, surgical resection status, and GRIm risk classification, was compared with the clinical model without GRIm, GRIm alone, and treat-all and treat-none strategies across a range of threshold probabilities. The full model demonstrated a favorable net benefit across most clinically relevant threshold probabilities.

Decision curve analysis was performed to further evaluate the potential clinical utility of the multivariate prediction models. For 3-year mortality risk, the full model incorporating clinical variables and GRIm risk classification showed a higher net benefit than the treat-all and treat-none strategies across a clinically relevant range of threshold probabilities (Figure 6). Similarly, for 3-year progression risk, the full model provided a favorable net benefit compared with the clinical model, GRIm alone, and default strategies across most threshold probabilities (Figure 7). These findings suggest that incorporating GRIm risk classification may improve the potential clinical usefulness of prediction models; however, this exploratory analysis requires external validation.

### 3.8. Sensitivity Analysis in the Resected Cohort

In a sensitivity analysis restricted to patients who underwent curative-intent surgical resection, postoperative ypStage was incorporated into the multivariate OS model. In this resected cohort, high GRIm risk classification remained independently associated with worse OS after adjustment for age, clinical stage, and postoperative ypStage (HR: 3.282, 95% CI: 1.361–7.916, *p* = 0.008). Advanced clinical stage showed a trend toward worse OS (HR: 2.919, 95% CI: 0.983–8.673, *p* = 0.054), whereas age was not independently associated with OS (HR: 1.044, 95% CI: 0.990–1.101, *p* = 0.115). Postoperative ypStage did not retain independent statistical significance in the multivariate OS analysis. Compared with pCR, the HRs for ypStage I, ypStage II, and ypStage III were 0.905 (95% CI: 0.137–5.968, *p* = 0.917), 2.882 (95% CI: 0.840–9.892, *p* = 0.093), and 2.391 (95% CI: 0.592–9.655, *p* = 0.221), respectively. The model showed an acceptable apparent discriminative performance, with a Harrell’s C-index of 0.793.

For PFS, the postoperative ypStage was incorporated into the multivariate model in the resected cohort. High GRIm risk classification remained independently associated with worse PFS after adjustment for age, histological subtype, clinical stage, and postoperative ypStage (HR: 6.718, 95% CI: 2.516–17.934, *p* < 0.001). Signet-ring-cell carcinoma was also independently associated with poorer PFS than adenocarcinoma (HR: 2.933, 95% CI: 1.153–7.461, *p* = 0.024). Clinical stage was not independently associated with PFS in this model (HR: 1.750, 95% CI: 0.672–4.556, *p* = 0.252). Postoperative ypStage was significantly associated with PFS (*p* = 0.045). Compared with pCR, the HRs for ypStage I, ypStage II, and ypStage III were 1.098 (95% CI: 0.138–8.731, *p* = 0.929), 2.283 (95% CI: 0.453–11.508, *p* = 0.317), and 5.849 (95% CI: 1.186–28.831, *p* = 0.030), respectively. The resected cohort PFS model showed good apparent discriminative performance, with a Harrell’s C-index of 0.811.

### 3.9. Additional and Exploratory Analyses

Additional descriptive analyses were performed in patients who underwent curative-intent surgical resection (*n* = 89), all of whom achieved R0 resection. Among these patients, 55 (61.8%) received adjuvant FLOT chemotherapy, and 42 (76.4%) of those who initiated adjuvant treatment completed all planned cycles. The pCR was associated with improved survival outcomes in the resected cohort. Patients who achieved pCR had a significantly longer median OS than those without pCR (73.8 vs. 53.4 months; log-rank *p* = 0.024). Similarly, pCR was associated with significantly improved PFS, with median PFS not reached in patients with pCR compared with 42.9 months in those without pCR (log-rank *p* = 0.001).

Exploratory nomograms based on the final multivariable Cox models were constructed for individualized estimation of 3-year OS and PFS probabilities. The OS nomogram incorporated age, clinical stage, surgical resection status, and GRIm risk classification, whereas the PFS nomogram included age, histological subtype, clinical stage, surgical resection status, and GRIm risk classification (Supplementary Figures S1 and S2). These nomograms were intended as visual representations of internally derived prediction models.

## 4. Discussion

This study evaluated the prognostic significance of the GRIm score in patients with locally advanced gastric or gastroesophageal junction adenocarcinoma who were treated with neoadjuvant FLOT chemotherapy. The results revealed that patients with a high GRIm risk had significantly worse survival outcomes and a higher risk of death and disease progression. In contrast, exploratory analyses using the cohort-specific ROC-derived NLR did not consistently show prognostic significance. The results of this study suggest that the use of composite indices, including inflammatory, nutritional, and metabolic parameters, can provide a more holistic prognostic assessment than the use of isolated inflammatory markers in the neoadjuvant setting.

In this group, the prognostic relevance of the GRIm score appears to be consistent with the increasing evidence supporting its importance in different solid tumors. The GRIm score combines three easily obtainable laboratory parameters (LDH, serum albumin, and NLR) into one index reflecting systemic inflammation and nutritional status [11,12]. Increased LDH levels have been associated with tumor volume, increased metabolic activity, and hypoxia-induced adaptations that may indicate more aggressive tumor behavior [5,26]. Conversely, low serum albumin levels may indicate malnutrition and an increased inflammatory response, both of which have been associated with poor outcomes in gastric cancer [5,27]. The NLR is an indicator of the balance between pro-tumor inflammatory processes and the antitumor response of the immune system [28,29,30]. However, its prognostic value may be limited when considered alone, as indicated by the present study [31,32,33]. Our results show that the relatively better prognostic ability of the GRIm score compared to that of the NLR alone may be attributed to the composite nature of the score. Exploratory analyses using the cohort-specific ROC-derived NLR cutoff in this cohort did not show a significant association with survival outcomes. This finding suggests that the individual components of the GRIm score may offer additional prognostic value compared to the NLR alone. The prognostic value of the GRIm score has been highlighted in previous meta-analyses, with a potential advantage over single inflammatory markers in different types of cancer [16,17]. The combination of LDH and albumin with NLR may improve the score’s ability to reflect the complex relationship between tumor burden, systemic inflammation, and nutritional status [5,7].

Surgical resection was significantly associated with improved survival, both OS and PFS, in our cohort. After neoadjuvant therapy, operability was an important factor for improving outcomes in patients who underwent surgery and had significantly better outcomes than those who did not. This is consistent with the established role of surgery as the cornerstone of curative treatment for locally advanced gastric cancer [22,34]. It is worth mentioning that patients undergoing surgery after neoadjuvant chemotherapy might belong to a more favorable group with a better response to therapy and general health status. Therefore, the observed survival advantage may be partially attributable to the selection bias inherent in retrospective studies. However, these findings emphasize the importance of careful patient selection and a multidisciplinary approach to improve treatment strategies.

In our study, we found that pCR was associated with a higher survival rate, which is consistent with previous studies. The large FLOT4 trial showed that patients who achieved pCR had better outcomes, with a complete pathological response rate of 16.6% in the FLOT group versus 6% in the ECF/ECC group [18,19]. In real-world studies, pCR has been associated with improved overall and disease-free survival in patients who received perioperative FLOT chemotherapy [35,36]. Kaplan–Meier analyses revealed a significant association between pCR achievement and improved OS and PFS in our cohort. In the resected cohort, postoperative ypStage was independently associated with PFS, with ypStage III disease having significantly worse PFS than pCR in the sensitivity analysis. However, postoperative ypStage was not an independent statistically significant factor for OS. These results are consistent with the concept that pathological response may be a surrogate of treatment efficacy and could better mirror short- to mid-term disease control, especially in terms of PFS.

In this cohort, clinical stage was independently associated with survival outcomes, emphasizing the known prognostic significance of TNM staging in gastric cancer. However, the clinical stage remains an important aspect of prognosis and treatment management, although molecular profiling and biomarker studies are constantly evolving [3]. However, the lack of consideration of tumor heterogeneity and host-related factors in the TNM staging system highlights the need for additional markers for improved risk stratification. In the current study, a high GRIm risk was independently associated with worse OS and PFS after adjusting for clinical stage, indicating that GRIm may provide additional risk stratification over the TNM stage alone [5,7].

Caution should be exercised in the association between signet-ring cell carcinoma and PFS. The prognostic significance of signet-ring cell histology in gastric cancer remains controversial, with studies reporting conflicting results based on the disease stage and patient populations studied [37,38,39]. Signet-ring cell carcinoma has been associated with a better prognosis in the early stages of the disease in some studies, possibly related to its tendency to remain localized to the mucosa and less common involvement of lymph nodes [38,39]. However, for advanced gastric cancers, many studies have demonstrated that this histological subtype may be associated with worse survival and higher early recurrence [40,41]. In our cohort of patients with locally advanced disease, signet-ring cell histology was associated with inferior PFS in the univariate analysis; however, this association was not significant in the multivariate models. These results may be due to the more aggressive biological nature of this subtype, including diffuse infiltration and peritoneal spread [39,41]. In the multivariate analysis, age was independently associated with OS and PFS. This is consistent with prior studies that indicated that the outcomes might not be as good with increasing age, likely because of the increased burden of comorbidities and decreased tolerance to treatment [42,43,44,45].

The GRIm score may provide additional prognostic information for the risk stratification of patients with locally advanced gastric or GEJ adenocarcinoma treated with neoadjuvant FLOT. Nevertheless, some traditional indices, including the Advanced Lung Cancer Inflammation Index (ALI), Prognostic Nutritional Index (PNI), and Controlling Nutritional Status (CONUT) score, have been demonstrated to have prognostic significance in gastric cancer, but they mainly reflect nutritional status or systemic inflammation as independent clinical domains [16,46]. In particular, PNI and CONUT are based on albumin and lymphocyte levels as indicators of nutritional risk, often ignoring complex metabolic-inflammatory interactions [46]. However, the GRIm score provides a more complete assessment by simultaneously including inflammatory, nutritional (albumin), and metabolic parameters at the same time [47]. The GRIm score incorporates LDH as an indicator of tumor burden and metabolic aggressiveness, thus reflecting the interaction between host physiology and tumor biology [16]. Similarly, recent studies on other solid tumors have proposed that composite metabolic-inflammatory indices, such as the glucose-to-CRP ratio (GCR), may yield prognostic information by combining systemic inflammation with metabolic alterations [48]. Thus, this integrative model may provide a more comprehensive prognostic assessment than traditional indices based on a single domain.

ROC analysis demonstrated that the a priori GRIm risk classification showed moderate discriminative ability and was better than the NLR alone. Although this level of discrimination may not be sufficient to be used as a single predictive tool, it may provide clinically useful information when incorporated into multivariable prognostic models or exploratory nomograms [49]. However, the exploratory cohort-specific ROC-derived NLR cutoff showed poor discriminative performance and was not consistently associated with survival outcomes in subsequent analyses. The limited performance of NLR in our cohort, despite its previous prognostic significance in gastric cancer [28,29,30], suggests that a single inflammatory marker is insufficient to reflect the complex biological heterogeneity of patients receiving neoadjuvant FLOT chemotherapy. These results suggest that the prognostic value of GRIm may not be due only to its inflammatory component but to the combined integration of inflammatory, nutritional, and metabolic parameters, including NLR, albumin, and LDH.

The feasibility of perioperative FLOT remains a significant clinical concern because of the intensive nature of the triplet regimen. In our cohort, 89 patients underwent curative-intent R0 resection, of whom 55 (61.8%) initiated adjuvant FLOT. Notably, 42 of the 55 patients (76.4%) completed all planned adjuvant cycles. This attrition rate underscores the inherent challenges of the postoperative phase, where treatment-related toxicities and a decline in performance status often impede adherence [50,51]. Similar results were observed in the pivotal FLOT4-AIO trial, where suboptimal adherence was found in the adjuvant setting, with only approximately 47% of patients completing the entire perioperative course [52]. Adjuvant completion rates in recent real-world studies have been reported to be between 29% and 56%, often related to postoperative complications, anorexia, and cumulative hematological toxicity [50,51]. Our results suggest that although the adjuvant phase is demanding, high completion rates are achievable in real-world practice through rigorous patient selection and proactive toxicity management.

The main multivariate models for overall survival and progression-free survival demonstrated good apparent discriminative performance, with Harrell’s C-index values of 0.831 and 0.859, respectively. Sensitivity analyses restricted to the resected cohort and incorporating postoperative ypStage also showed acceptable-to-good apparent discrimination, with C-index values of 0.793 and 0.811 for OS and PFS, respectively. In addition, calibration analyses for 3-year OS and PFS showed acceptable apparent agreement between the model-predicted and Kaplan–Meier-estimated observed survival probabilities. Exploratory nomograms and decision curve analyses were also performed to visualize individualized risk prediction and assess the potential clinical net benefits of the prognostic models. However, these findings should be interpreted cautiously, as discrimination, calibration, nomogram construction, and decision curve analysis were all internally derived from the same retrospective cohort, and external validation was not performed.

The strength of this study is the relatively homogeneous cohort of patients, all of whom were treated with a standardized neoadjuvant FLOT regimen and followed up in a real-world clinical setting. A uniform treatment protocol probably diminished treatment-related variability, thus facilitating a more reliable evaluation of prognostic factors. In addition, the inclusion of a wide range of clinical, laboratory, and pathological variables, including inflammatory and nutritional parameters, allowed a more complete analysis of the factors associated with survival within this treatment framework.

This study had several limitations. First, its retrospective and single-center nature might restrict its generalizability and introduce selection bias, preventing causal conclusions from being drawn. Second, the relatively small sample size may have diminished statistical power, especially in subgroup analyses. Third, differences in treatment exposure, as not all patients underwent surgical resection or completed the planned adjuvant chemotherapy, might have affected survival outcomes and added variability. Although adjuvant chemotherapy administration and completion rates were reported descriptively, a definitive survival analysis according to adjuvant chemotherapy completion status was not performed because of the limited sample size and potential selection bias in the resected subgroup of patients. Fourth, differences in subsequent treatment lines and the absence of detailed information regarding recurrence patterns, metastatic burden, and treatment responses after progression may have contributed to the outcome variability, particularly for OS. Fifth, the GRIm score was evaluated only at baseline, and dynamic changes during treatment were not assessed. In addition, an unresolved methodological limitation is related to the NLR threshold used in the GRIm score. In the present study, the original predefined NLR cutoff of ≥6 was retained to maintain consistency with the original GRIm scoring system and to allow comparability with previous studies. However, the cohort-specific ROC-derived NLR cutoff was lower, and the use of the predefined threshold may have introduced a stratification bias by potentially misclassifying some patients. Therefore, the prognostic value of the GRIm score in this setting should be interpreted as exploratory and requires external validation in larger, independent cohorts. Sixth, another important unresolved limitation is the absence of detailed surgical metrics. Although curative-intent surgery was performed according to institutional practice and contemporary gastric cancer surgical guidelines whenever feasible, data regarding the extent of lymphadenectomy, including D1 versus D2 dissection and lymph node yield, were not consistently available in the medical records. Because these variables may significantly influence survival outcomes, their absence limits the ability to adjust for surgical quality and reduces the clinical generalizability of our findings. Finally, the lack of molecular data, including human epidermal growth factor receptor 2 (HER2), microsatellite instability (MSI), and programmed death ligand 1 (PD-L1), limits the evaluation of the GRIm score within modern biomarker-driven treatment strategies [19,20].

## 5. Conclusions

In conclusion, the GRIm score was associated with OS and PFS in patients with locally advanced gastric cancer who were treated with neoadjuvant FLOT chemotherapy. The GRIm score, which combines information on systemic inflammation and nutritional status, may provide additional prognostic value compared to conventional clinicopathological factors, including clinical stage, pathological response, and feasibility of curative-intent surgery. However, the interpretation of these results should be cautious, as this was a single-center retrospective study and two unresolved methodological limitations still exist. First, in this cohort, stratification bias may have been introduced by the predefined NLR threshold used in the original GRIm score. Second, detailed surgical quality metrics, including the status of D1/D2 lymphadenectomy and lymph node yield, were not consistently available. Therefore, the clinical generalizability of these findings is still limited, and further prospective multicenter studies are warranted to validate the prognostic relevance of the GRIm score prior to any routine clinical application.

## Figures and Tables

**Table 1:** Baseline clinicopathological characteristics (*n* = 128).

Variable	*n* (%)/Mean ± SD
Age (years)	61.46 ± 9.01
Sex	
Male	81 (63.3)
Female	47 (36.7)
Smoking	53 (41.4)
ECOG Performance Status	
0	28 (21.9)
1	100 (78.1)
Comorbidity	49 (38.3)
Diabetes mellitus	10 (7.8)
Hypertension	21 (16.4)
Coronary artery disease	13 (10.2)
COPD	2 (1.6)
Other	3 (2.3)
Histological Type	
Adenocarcinoma	72 (56.3)
Signet ring cell carcinoma	56 (43.8)
Tumor Location	
GEJ	18 (14.1)
Gastric body	87 (68.0)
Antrum/Pylorus	23 (18.0)
Clinical T Stage	
T2	29 (22.7)
T3	83 (64.8)
T4a	14 (10.9)
T4b	2 (1.6)
Clinical N Stage	
N0	39 (30.5)
N1	28 (21.9)
N2	32 (25.0)
N3	29 (22.7)
Clinical Stage	
Stage I	11 (8.6)
Stage II	46 (35.9)
Stage III	69 (53.9)
Stage IVA	2 (1.6)

Abbreviations: ECOG, Eastern Cooperative Oncology Group; COPD, chronic obstructive pulmonary disease; GEJ, gastroesophageal junction; SD, standard deviation.

**Table 2:** Treatment characteristics and clinical outcomes of the study cohort.

Variable	*n* (%)/Mean ± SD
Surgery	89 (69.5)
pCR	22 (24.7) *
ypT Stage (*n* = 89)	
T0	22 (24.7)
T1	5 (5.6)
T2	12 (13.5)
T3	36 (40.4)
T4a	14 (15.7)
ypN Stage (*n* = 89)	
N0	52 (58.4)
N1	10 (11.2)
N2	12 (13.5)
N3	15 (16.9)
Postoperative Stage (*n* = 89)	
pCR	22 (24.7) *
Stage I	13 (14.6)
Stage II	25 (28.1)
Stage III	29 (32.6)
Progression	73 (57.0) **
Dead	62 (48.4) **

Abbreviations: pCR, pathological complete response; ypT, post-treatment pathological tumor stage; ypN, post-treatment pathological nodal stage; SD, standard deviation. * pCR rate calculated among patients who underwent surgical resection (*n* = 89). ** Percentages calculated based on the total study cohort.

**Table 3:** Laboratory parameters and GRIm score (*n* = 128).

Variable	*n* (%)/Mean ± SD
Neutrophils (×10^9^/L)	5.04 ± 1.91
Lymphocytes (×10^9^/L)	2.15 ± 0.75
NLR	2.75 ± 1.82
LDH (U/L)	233.25 ± 63.69
Albumin (g/dL)	3.81 ± 0.39
GRIm Score	
0	65 (50.8)
1	26 (20.3)
2	31 (24.2)
3	6 (4.7)
GRIm Risk Group	
Low risk	91 (71.1)
High risk	37 (28.9)

Abbreviations: NLR, neutrophil-to-lymphocyte ratio; LDH, lactate dehydrogenase (U/L); GRIm score, Gustave Roussy Immune score; SD, standard deviation; g/dL, grams per deciliter; ×10^9^/L, cells per liter.

**Table 4:** Baseline clinicopathological characteristics according to GRIm risk classification.

Variable	Low-Risk (*n* = 91)	High-Risk (*n* = 37)	*p*-Value
Age (years)	60.6 ± 9.2	63.6 ± 8.3	0.092
Sex			0.521
Male	56 (61.5%)	25 (67.6%)	
Female	35 (38.5%)	12 (32.4%)	
ECOG Performance Status			0.606
0	21 (23.1%)	7 (18.9%)	
1	70 (76.9%)	30 (81.1%)	
Comorbidity			0.948
Absent	56 (61.5%)	23 (62.2%)	
Present	35 (38.5%)	14 (37.8%)	
Histological Type			0.210
Adenocarcinoma	48 (52.7%)	24 (64.9%)	
Signet-ring cell carcinoma	43 (47.3%)	13 (35.1%)	
Tumor Location			0.795
Gastroesophageal junction	14 (15.4%)	4 (10.8%)	
Gastric body	61 (67.0%)	26 (70.3%)	
Antrum/Pylorus	16 (17.6%)	7 (18.9%)	
Clinical T Stage			0.187
T2	23 (25.3%)	6 (16.2%)	
T3	59 (64.8%)	24 (64.9%)	
T4a	7 (7.7%)	7 (18.9%)	
T4b	2 (2.2%)	0 (0.0%)	
Clinical N Stage			0.071
N0	31 (34.1%)	8 (21.6%)	
N1	23 (25.3%)	5 (13.5%)	
N2	21 (23.1%)	11 (29.7%)	
N3	16 (17.6%)	13 (35.1%)	
Clinical Stage			0.079
Stage I–II	45 (49.5%)	12 (32.4%)	
Stage III–IV	46 (50.5%)	25 (67.6%)	
Surgery			0.114
No	24 (26.4%)	15 (40.5%)	
Yes	67 (73.6%)	22 (59.5%)	

Abbreviations: ECOG, Eastern Cooperative Oncology Group; GRIm score, Gustave Roussy Immune score.

**Table 5:** Univariate and multivariate Cox regression analysis for overall survival.

Variable	Univariate HR (95% CI)	*p*-Value	Multivariate HR (95% CI)	*p*-Value
Age	1.045 (1.013–1.077)	0.005	1.035 (1.007–1.064)	0.013
Sex	0.891 (0.526–1.509)	0.668	-	-
Histological type *	1.436 (0.844–2.443)	0.182	-	-
Clinical stage (III–IV vs. I–II)	2.619 (1.490–4.605)	<0.001	2.257 (1.258–4.050)	0.006
Exploratory NLR analysis (≥3.43 vs. <3.43)	1.463 (0.833–2.569)	0.185	-	-
Surgery (Yes vs. No)	0.137 (0.080–0.233)	<0.001	0.164 (0.095–0.285)	<0.001
GRIm risk (High vs. Low)	3.099 (1.869–5.138)	<0.001	1.832 (1.059–3.169)	0.030

Abbreviations: HR, hazard ratio; CI, confidence interval; NLR, neutrophil-to-lymphocyte ratio; GRIm score, Gustave Roussy Immune score. * Adenocarcinoma was the reference category for histological type.

**Table 6:** Univariate and multivariate Cox regression analysis for progression-free survival.

Variable	Univariate HR (95% CI)	*p*-Value	Multivariate HR (95% CI)	*p*-Value
Age	1.029 (1.002–1.058)	0.038	1.050 (1.022–1.078)	<0.001
Sex	0.909 (0.560–1.475)	0.699	—	—
Histological type *	1.661 (1.048–2.632)	0.031	1.517 (0.926–2.485)	0.098
Clinical stage (III–IV vs. I–II)	1.896 (1.166–3.081)	0.010	1.722 (1.028–2.883)	0.039
Exploratory NLR analysis (≥3.43 vs. <3.43)	1.572 (0.922–2.679)	0.096	—	—
Surgery (Yes vs. No)	0.037 (0.020–0.069)	<0.001	0.039 (0.020–0.076)	<0.001
GRIm risk (High vs. Low)	2.874 (1.798–4.593)	<0.001	2.491 (1.483–4.185)	<0.001

Abbreviations: HR, hazard ratio; CI, confidence interval; NLR, neutrophil-to-lymphocyte ratio; GRIm score, Gustave Roussy Immune score. * Adenocarcinoma was used as the reference category for histological type.

## Data Availability

The datasets generated and/or analyzed during the current study are not publicly available due to patient privacy and institutional data protection policies but are available from the corresponding author upon reasonable request.

## Supplementary Materials

The following supporting information can be downloaded at: https://www.mdpi.com/article/10.3390/diagnostics16121759/s1, Figure S1. Nomogram for predicting 3-year overall survival. The nomogram was constructed based on the final multivariable Cox regression model for overall survival, including age, clinical stage, surgical resection status, and GRIm risk classification. Each variable was assigned a point value according to its relative prognostic contribution, and the total points corresponded to the estimated probability of 3-year overall survival. This nomogram should be interpreted as an exploratory visual representation of an internally derived prediction model. Figure S2. Nomogram for predicting 3-year progression-free survival. The nomogram was constructed based on the final multivariable Cox regression model for progression-free survival, including age, histological type, clinical stage, surgical resection status, and GRIm risk classification. Each variable was assigned a point value according to its relative prognostic contribution, and the total points corresponded to the estimated probability of 3-year progression-free survival. This nomogram should be interpreted as an exploratory visual representation of an internally derived prediction model.

## Abbreviations

The following abbreviations are used in this manuscript.

AJCC	American Joint Committee on Cancer
ALI	Advanced Lung Cancer Inflammation Index
AUC	Area under the curve
C-index	Concordance index
cTNM	Clinical tumor–node–metastasis
CI	Confidence interval
CONUT	Controlling Nutritional Status
CT	Computed tomography
DCA	Decision curve analysis
ECOG	Eastern Cooperative Oncology Group
FLOT	Fluorouracil, leucovorin, oxaliplatin, and docetaxel
GCR	Glucose-to-C-reactive protein ratio
GEJ	Gastroesophageal junction
GRIm	Gustave Roussy Immune
HER2	Human epidermal growth factor receptor 2
HR	Hazard ratio
IQR	Interquartile range
LDH	Lactate dehydrogenase
MSI	Microsatellite instability
NLR	Neutrophil-to-lymphocyte ratio
OS	Overall survival
pCR	Pathological complete response
PD-L1	Programmed death-ligand 1
PET-CT	Positron emission tomography–computed tomography
PFS	Progression-free survival
PNI	Prognostic Nutritional Index
RECIST	Response Evaluation Criteria in Solid Tumors
ROC	Receiver operating characteristic
SD	Standard deviation
TNM	Tumor–Node–Metastasis
ypN	Pathological nodal stage after neoadjuvant therapy
ypStage	Pathological stage after neoadjuvant therapy
ypT	Pathological tumor stage after neoadjuvant therapy
ypTNM	Pathological tumor–node–metastasis after neoadjuvant therapy
