# Supplementary figures and images for: Prognostic Value of the Gustave Roussy Immune Score in Patients with Locally Advanced Gastric Cancer Receiving Neoadjuvant FLOT Chemotherapy: A Retrospective Cohort Study
Source: Diagnostics (Basel). 2026 Jun 7;16(12):1759. doi: 10.3390/diagnostics16121759 (PMC13298714; doi:10.3390/diagnostics16121759)

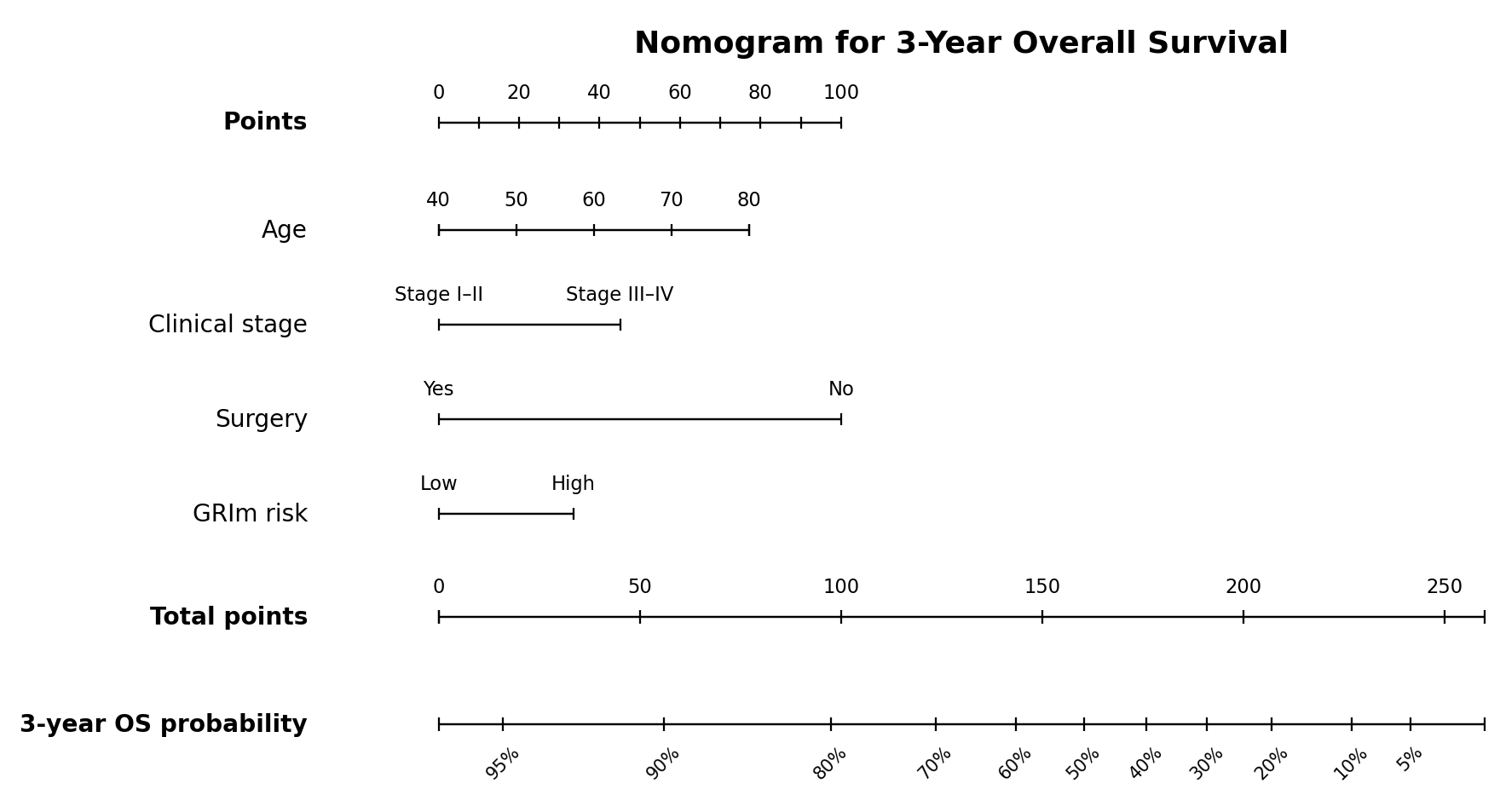

Supplement: Supplementary file 1 [file diagnostics-16-01759-s001.zip › Figure_S1_OS_3year_nomogram_corrected.png]

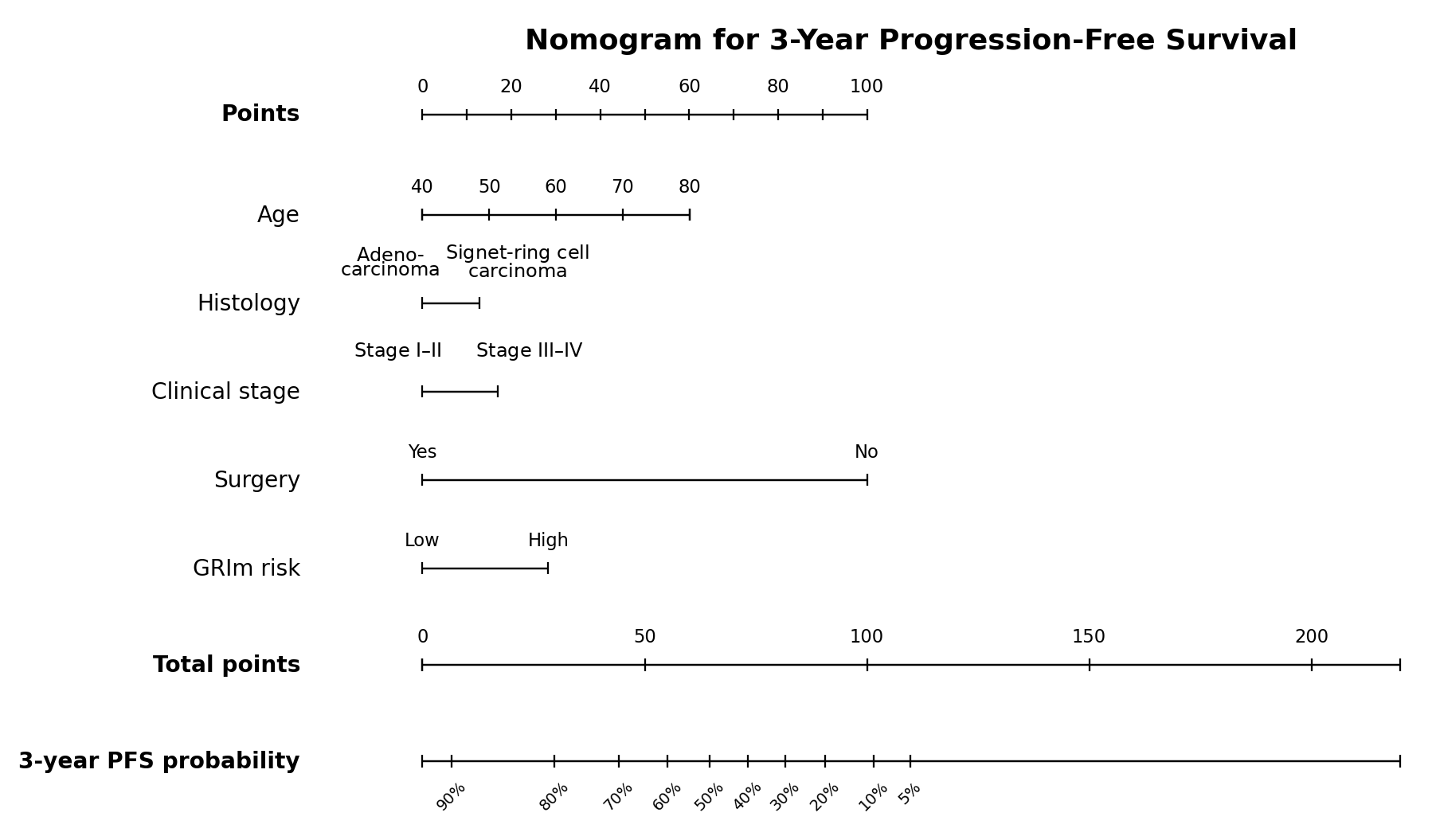

Supplement: Supplementary file 1 [file diagnostics-16-01759-s001.zip › Figure_S2_PFS_3year_nomogram_final_stage_font_matched.png]
